# CD133阳性/阴性肺癌细胞的分选、鉴定及差异基因的筛选

**DOI:** 10.3779/j.issn.1009-3419.2015.03.01

**Published:** 2015-03-20

**Authors:** 少秋 郑, 书华 李, 红艳 王, 晓斌 谢, 雅洁 张

**Affiliations:** 1 514031 梅州，梅州市人民医院病理科 Department of Pathology, Meizhou People's Hospital, Meizhou 514031, China; 2 510182 广州，广州医科大学病理教研室 Department of Pathology, Guangzhou Medical University, Guangzhou 510182, China

**Keywords:** CD133, 肺腺癌, 磁式分选, 基因芯片, 转移相关基因, CD133, Adenocarcinoma of lung, Magnetic activated cell sorting (MACS), DNA microarray, Metastasis-related genes

## Abstract

**背景与目的:**

研究表明多种实体肿瘤中存在肿瘤干细胞（cancer stem cells, CSCs），肿瘤干细胞与非肿瘤干细胞的生物学特性存在已有的明显差异，而CD133被认为是肿瘤干细胞的标记物，因此CD133阳性细胞与CD133阴性细胞可能存在明显差异。本研究通过分选人肺腺癌A549细胞中的CD133阳性及CD133阴性细胞，鉴定两组细胞的生物学特性并在人组织标本进行验证，利用基因芯片筛选转移相关差异基因。

**方法:**

采用磁式分选（magnetic activated cell sorting, MACS）的方法对人肺腺癌A549中CD133阳性细胞、CD133阴性细胞进行分选，通过无血清条件培养、平板克隆、血清诱导分化及基因芯片等实验，比较两组细胞“sphere”形成、细胞增殖、细胞分化以及差异基因，并在人组织标本进行CD133表达与临床特性的验证分析。

**结果:**

CD133阳性细胞能在无血清培养基中悬浮生长并形成“sphere”；其平均克隆形成率（57.1%）明显高于CD133阴性细胞（3.3%）；CD133阳性细胞能被血清诱导分化、表达腺癌标志物CK7；两组细胞中的19个转移基因表达水平相差两倍或以上，差异最高达12倍；肺癌组织中CD133阳性细胞主要分布于癌巢周边，数量稀少，其表达与肿瘤组织学分型、分级及临床分期无关。

**结论:**

CD133阳性肺腺癌A549细胞具有CSCs特性；两组细胞在转移相关基因的表达存在明显差异，其中CD82可能在CD133阳性细胞的转移机制中起重要作用。

## Abstract (en)

**Background and objective:**

It has been proven that cancer stem cell existed in variety of cancer, which an significant difference of biological characteristics was observed between the cancer stem cells and non-cancer stem cells. And CD133 is considered to be cancer stem cell marker. So there may be significant differences in CD133- positive cells and CD133-negative cells. The aim of this study is to isolate CD133+ cells and CD133- cells from lung cancer cell line A549, explore their biological characteristics and screen the metastasis-related genes.

**Methods:**

MACS was applied to isolate CD133+ cells and CD133- cells from human lung cancer cell line A549. To observe the formation of sphere, CD133+ cells and CD133- cells were cultured in serum-free DMEM-F12 medium (containing EGF, bFGF) *in vitro*. The colony formatting efficiency of CD133+ cells, CD133- cells and cells without sorting was tested by colony-forming assay. The differentiation of sphere was induced by culturing in DMEM-F12 medium (containing serum). The metastasis-related genes (84 genes) of CD133+ cells and CD133- cells were detected by using DNA microarray. Immunohistochemistry was used to detect the expression of CD133 protein in Human lung cancer tissue.

**Results:**

CD133+ cells formed sphere in serum-free DMEM-F12 medium, while the CD133- cells failed to form sphere. The rates of CD133+ cell colony formation (57.1%) was significantly higher than that of CD133- cells (3.3%). Sphere (CD133+/CK7-) was induced to differentiate, and CK7 expression was found in differentiated cells. The expression levels of 19 metastasis-related genes from CD133+ cells and CD133- cells were significant different. Little CD133 positive cells which distributing around the cancer nests were found in lung cancer tissue. The expression of CD133 was not related to tumor types, cell differentiation or TNM stage.

**Conclusion:**

CD133+ cells exhibit the characteristics of cancer stem cells. The difference of metastasis-related gene expression levels was discovered between CD133+ cells and CD133- cells. CD82 plays an important role in mechanism of tumor metastasis.

肿瘤转移和复发是恶性肿瘤临床治疗失败及患者死亡的主要原因，肿瘤干细胞（cancer stem cells, CSCs）是导致肿瘤发生转移、复发的关键所在^[1]^。

目前，人们已经分离出乳腺癌干细胞，并证实脑良性和恶性肿瘤、恶性黑色素瘤细胞系、结肠癌、肝癌、前列腺癌等实体瘤中存在CSCs^[2-4]^。肺癌是发病率和死亡率增长最快的恶性肿瘤，研究^[5, 6]^表明肺癌中存在CSCs，CD133可能是肺癌干细胞的标记物。本研究拟通过MACS的方法对肺癌细胞株A549中的CD133阳性细胞及CD133阴性细胞进行分选，并对其生物学特性进行鉴定。在此基础上，利用基因芯片对CD133阳性/阴性细胞进行对比检测，筛选转移相关的差异基因，旨在为肿瘤转移机制的研究提供新线索，为肺癌的基因治疗提供新靶点。

## 1 材料和方法

### 1.1 组织标本

收集广州医学院第一附属医院自2005年1月-2008年12月手术切除的81例肺癌石蜡包埋组织（患者术前均未接受化疗和放疗），患者年龄最大81岁，最小29岁，中位年龄50岁。根据2004年世界卫生组织肺癌组织分类标准进行分类，81例肺癌组织中鳞癌29例，腺癌34例，大细胞癌、腺鳞癌、肉瘤样癌各4例，小细胞癌6例；临床分期Ⅰ期28例，Ⅱ期21例，Ⅲ期27例，Ⅳ期5例；伴淋巴结转移者43例，无转移者38例。同时收集10例正常肺组织。

### 1.2 细胞培养

肺腺癌A549细胞系来源于ATCC，引自广州医学院中心实验室。采用含10%新生牛血清（Gibco）RPMI-1640培养液（Gibco）于37 ℃、5%CO_2_及饱和湿度培养箱中培养，每3天更换培养液。细胞密度为80%-100%时，0.25%胰酶-EDTA消化液消化后，1:3传代。

### 1.3 细胞分选

分别采用阳性分选柱（MS柱）和阴性分选柱（LD柱）分选CD133阳性细胞及CD133阴性细胞：A549细胞在75 cm^3^培养瓶中生长达80%融合时，0.25%胰酶EDTA消化液消化后，收获单细胞悬液，计数细胞，取≤10^8^个细胞过柱进行分选，步骤按试剂盒（Miltenyi）说明书进行。

### 1.4 免疫荧光技术检测细胞纯度

取经分选细胞（CD133阳性细胞、CD133阴性细胞）制备细胞涂片，利用PE标记CD133-2（Miltenyi）抗体进行免疫荧光检测，步骤按试剂盒说明进行。荧光显微镜观察，细胞呈现明亮红色荧光为阳性。

### 1.5 “sphere”形成实验及诱导分化实验

取等量的CD133阳性细胞及CD133阴性细胞按1, 000个/mL的比例置于添加了10 ng/mL碱性成纤维细胞生长因子，20 mg/mL表皮生长因子，50 mg/mL胰岛素，100 mg/mL脱铁转铁蛋白，10 mg/mL腐胺，0.03 mmol/L亚硒酸钠，2 mmol/L黄体酮，0.6%葡萄糖，5 mmol/L 4-羟乙基哌嗪乙磺酸，1%碳酸氢钠，0.4%牛血清白蛋白及谷氨酰胺的无血清DMEM-F12培养基中进行培养，待培养基变黄进行换液；取培养形成的“sphere”加入含10%胎牛血清（Gibco）的DMEM-F12（Gibco）培养基中，于37 ℃、5%CO_2_及饱和湿度培养箱培养，待细胞贴壁或培基变黄进行换液。

### 1.6 免疫组织化学（immunohistochemistry, IHC）

采用二步法进行CD133、CK7染色，步骤按试剂盒说明进行。对照设置：用PBS代替一抗作为阴性对照，选已知阳性病例作阳性对照。肿瘤细胞胞浆与胞膜呈现棕褐色为阳性。

### 1.7 平板克隆实验

选用12孔板，CD133阳性细胞、CD133阴性细胞、未分选细胞单细胞悬液（50个/孔）加入含10%胎牛血清RPMI-1640培养基，置于37 ℃、5%CO_2_培养箱中培养，待出现肉眼可见的细胞克隆时终止培养，显微镜观察并计数大于50个细胞的克隆数，然后计算克隆形成率。克隆形成率（%）＝（克隆数/接种细胞数）×100%。

### 1.8 基因芯片检测转移相关差异基因

采用SuperArray第二代功能分类基因芯片对CD133阳性细胞及CD133阴性细胞（原代）分别进行肿瘤转移基因（84个转移相关基因）的对比检测，结合生物信息学技术分析两组之间的差异基因。按常规方法提取CD133阳性细胞（实验组）及CD133阴性细胞（对照组）的RNA，合成cDNA、荧光定量PCR（real-time fluorescence quantitative PCR, RTFQ PCR）操作分别按照RT2 PCR Array First Strand Kit-C03、PCR Array试剂盒说明书进行操作。数据分析采用ΔΔCt方法。

### 1.9 统计学方法采用SPSS

13.0统计分析软件进行处理。两样本构成比（率）比较采用χ^2^检验，两组间均数比较采用*t*检验，多组间均数比较用单因素方差分析（Student Newman Keuls, SNK）。以*P*＜0.05为差异有统计学意义。

## 2 结果

### 2.1 IHC结果

肺癌组织中CD133阳性肿瘤细胞呈分散或小灶状/巢状分布于癌巢边缘，数量稀少。81例肺癌标本CD133表达阳性率为53.1%（43/81），CD133表达与各组织学类型的关系分析显示：鳞癌、腺癌及其他类型阳性率分别为48.3%（14/29）、52.9%（18/34）和61.1%（11/18），CD133的表达与组织学类型无关（*P*＞0.05）（表 1，图 1A-图 1B）；与肿瘤分级及临床分期无关（*P*＞0.05）。10例正常肺组织中均未见CD133阳性细胞。人肺腺癌A549细胞株中有CD133阳性细胞存在，数量稀少（图 2）。

**1:** CD133表达与临床病理特征的关系 Relationship between the expression of CD133 and clinicopathological features of lung cancer

Group	CD133		*P*
	Positive	Negative	
Histological type			> 0.05
Squamous cell carcinoma	14	15	
Adenocarcinoma	18	16	
The others^*^	11	7	
Pathological grading			> 0.05
Well-differentiated/ Moderately differentiated	19	20	
Poorly-differentiated	13	11	
Clinical stage			> 0.05
Ⅰ	14	14	
Ⅱ	12	9	
Ⅲ+Ⅳ	17	15	
^*^The others include small cell anaplastic carcinoma, large cell anaplastic carcinoma, mixed adenocarcinoma/squamous carcinoma, *et al*.			

**1:**
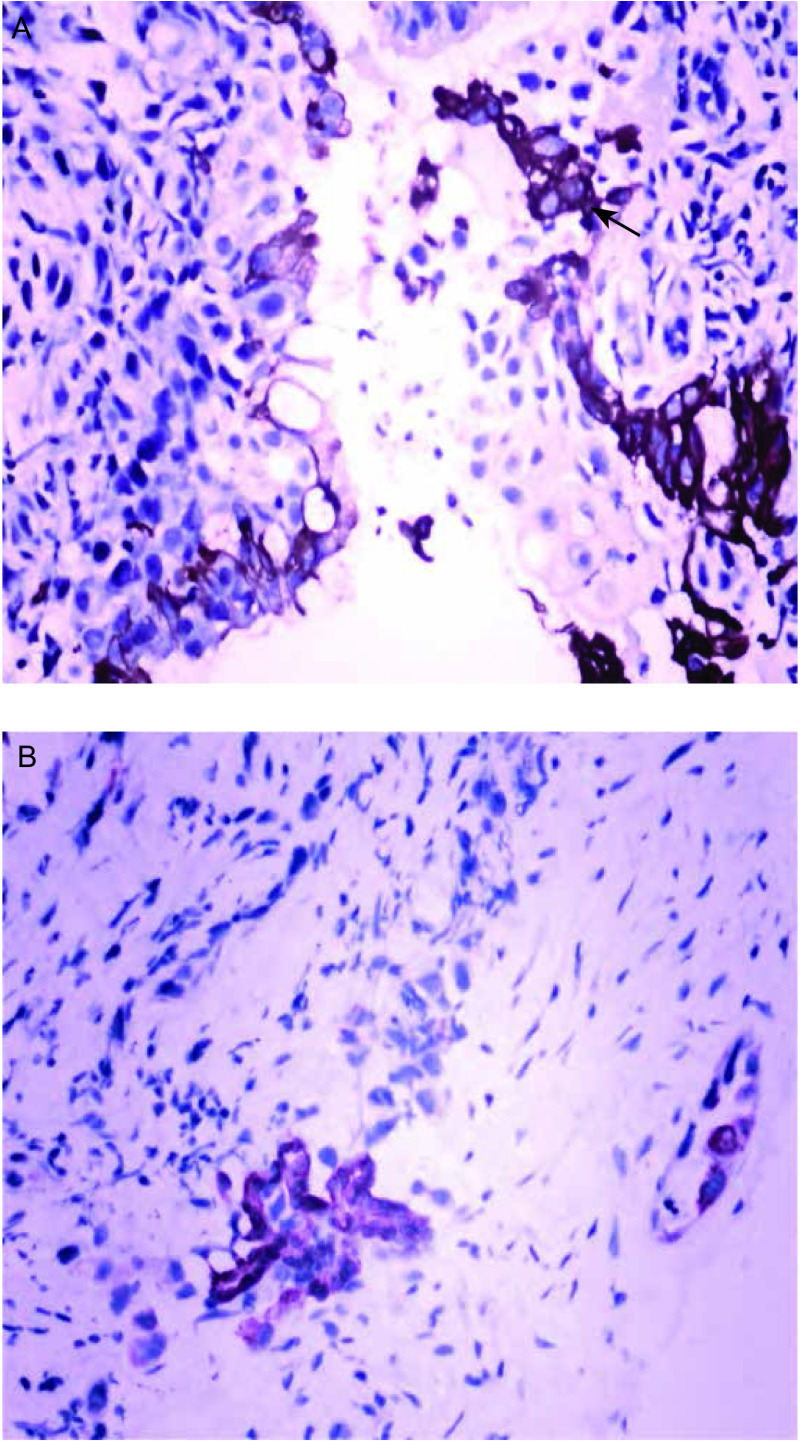
人肺癌组织中CD133阳性表达。A：肺鳞癌二步法（EnVision，×200）；B：肺腺癌二步法（EnVision，×200） CD133 expression of Human lung cancer tissues. A: Lung squamous cell carcinoma (EnVision, ×200); B: Lung adenocarcinoma (EnVision, ×200).

**2:**
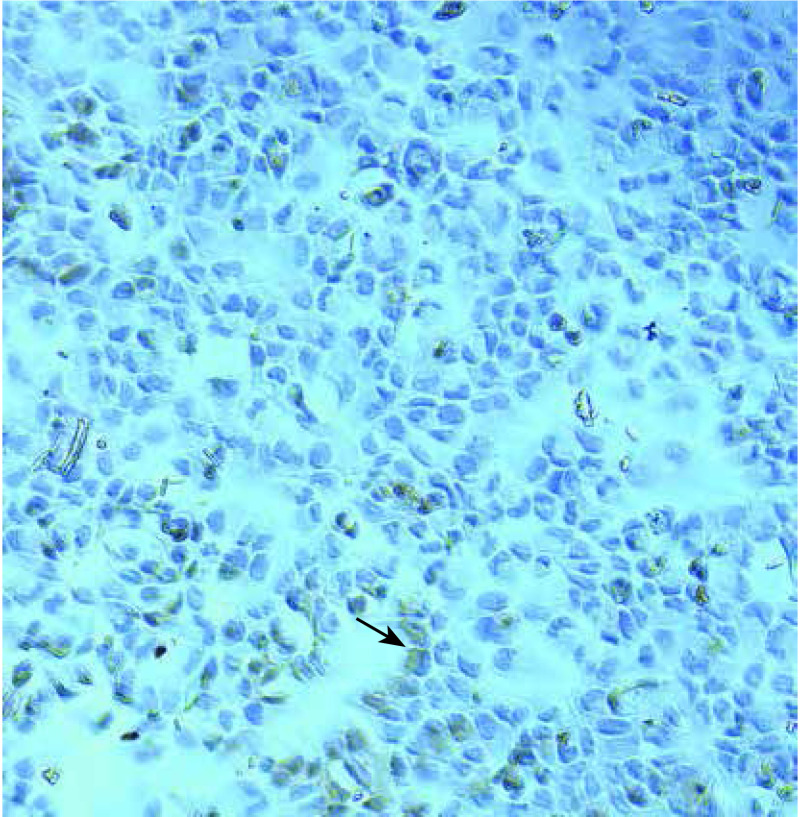
人肺腺癌A549细胞株中CD133阳性表达（EnVision，×200） CD133 expression of human lung cancer cell line A549 (EnVision, ×200)

### 2.2 MACS分选结果

从人肺腺癌A549细胞株中分选出CD133阳性细胞比例约为0.2%。分选获得的CD133阳性细胞中绝大部分细胞发明亮红色荧光（图 3），CD133阴性细胞中仅见少量细胞发微弱红色荧光，表明MACS分选所获得细胞纯度较高。

**3:**
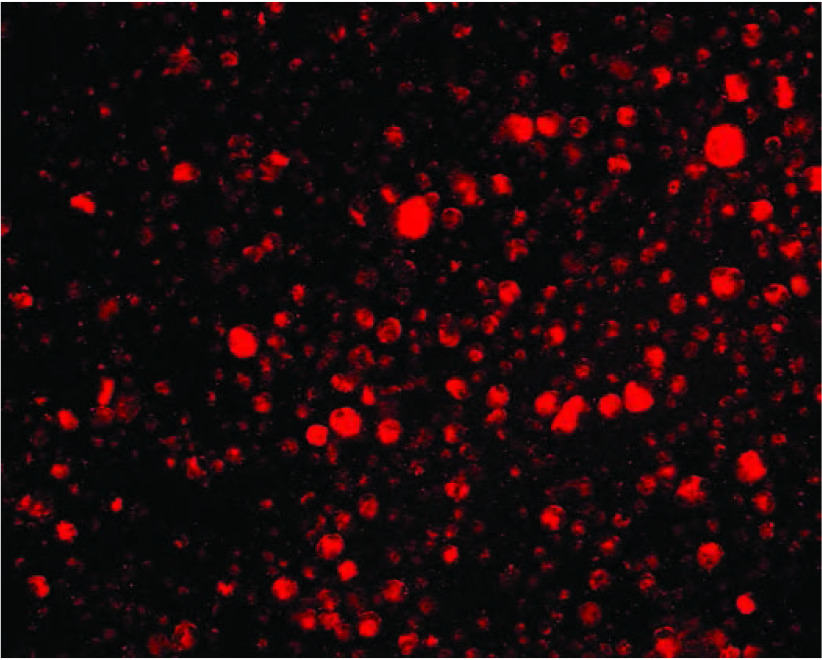
CD133阳性细胞（20×10，荧光显微镜） CD133-positive cells (20×10, Fluorescence microscopy)

### 2.3 “sphere”形成及诱导分化实验结果

CD133阳性细胞在无血清培养基中培养形成“sphere”，CD133阴性细胞培养8周未见“sphere”形成；“sphere”加入含血清培养基中培养呈现贴壁生长，诱导分化前表达CD133、不表达CK7；诱导分化后CD133阴性、CK7阳性（图 4）。

**4:**
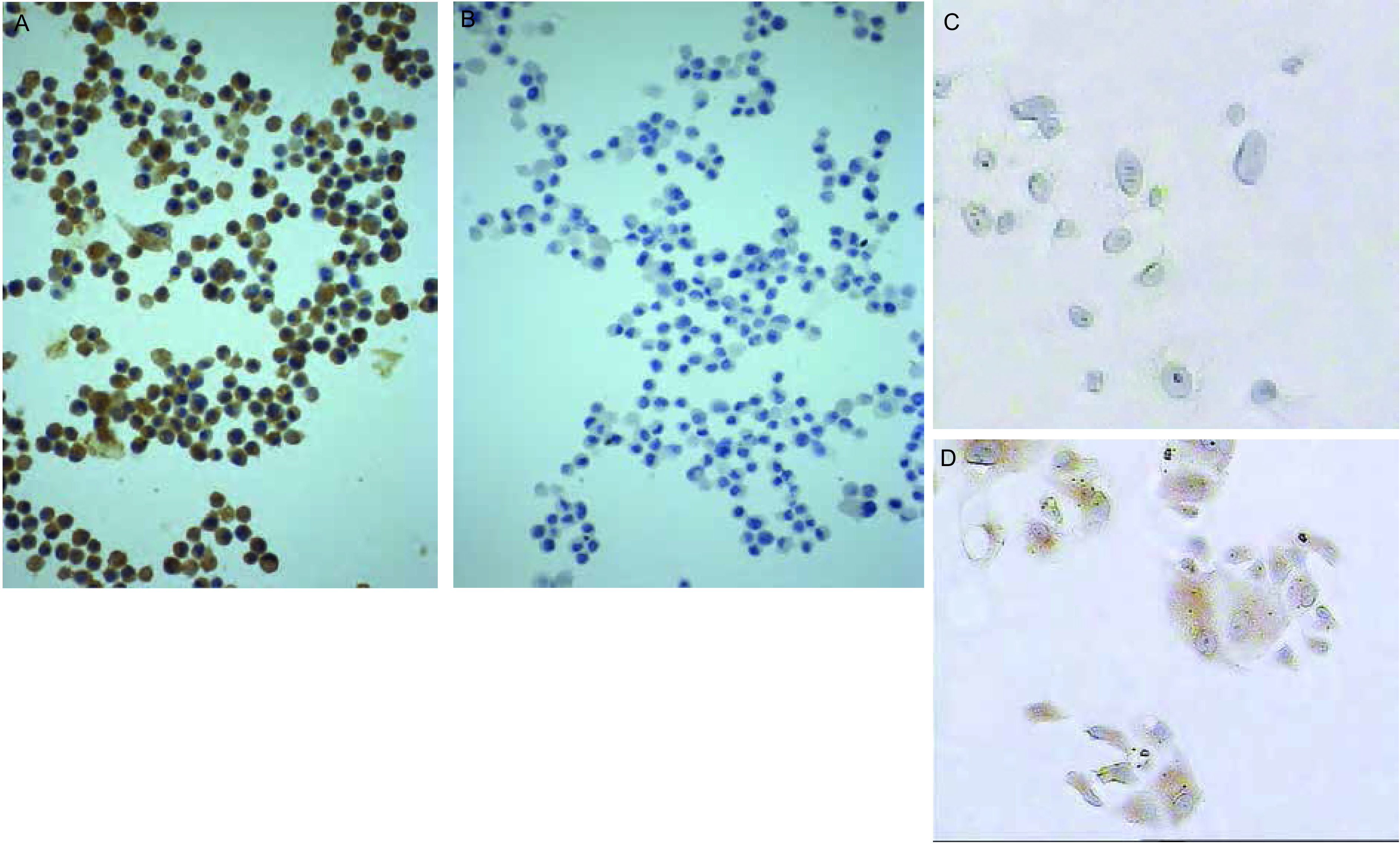
诱导分化前（A: CD133+, B: CK7-）及诱导分化后（C: CD133-, D: CK7+） Undifferentiated cells (A: CD133+, B: CK7-) & Differentiated cells (C: CD133-, D: CK7+)

### 2.4 平板克隆形成实验结果

CD133阳性细胞、CD133阴性细胞、未分选细胞组平均克隆形成率分别为57.1%、3.3%、8.7%，三组间克隆形成率差异有统计学意义（*P*＜0.05）（表 2）。

**2:** CD133阳性、CD133阴性细胞及未分选细胞的克隆形成率 The cloning forming rates of CD133-positive cells, CD133-negative cells and unsorted cells

Group	The cloning forming rates		
	The first hole	The second hole	The third hole
CD133-positive cells	52.0%	44.0%	76.0%
CD133-negative cells	2.0%	2.0%	6.0%
Unsorted cells	10.0%	8.0%	8.0%

### 2.5 RNA提取

核酸定量仪检测各组细胞总RNA的OD_260_/OD_280_均在1.7-2.0之间，说明RNA纯度高；变性琼脂糖凝胶电泳显示28s、18s条带清晰，前者荧光强度约为后者的两倍，5s条带弱，说明RNA完整、无降解。

### 2.6 基因芯片检测结果

显示从扩增曲线可见所有样品均已进入平台期，说明反应条件设定准确（图 5）。CD133阳性细胞和CD133阴性细胞的基因表达有显著差异。84个转移基因中，差异达两倍或以上的有19个，其中差异＞2倍、≤3倍的基因有14个，差异＞3倍、≤4倍的基因有3个，差异＞4倍、≤5倍的基因有1个，差异＞5倍的基因有1个（表 3）。

**5:**
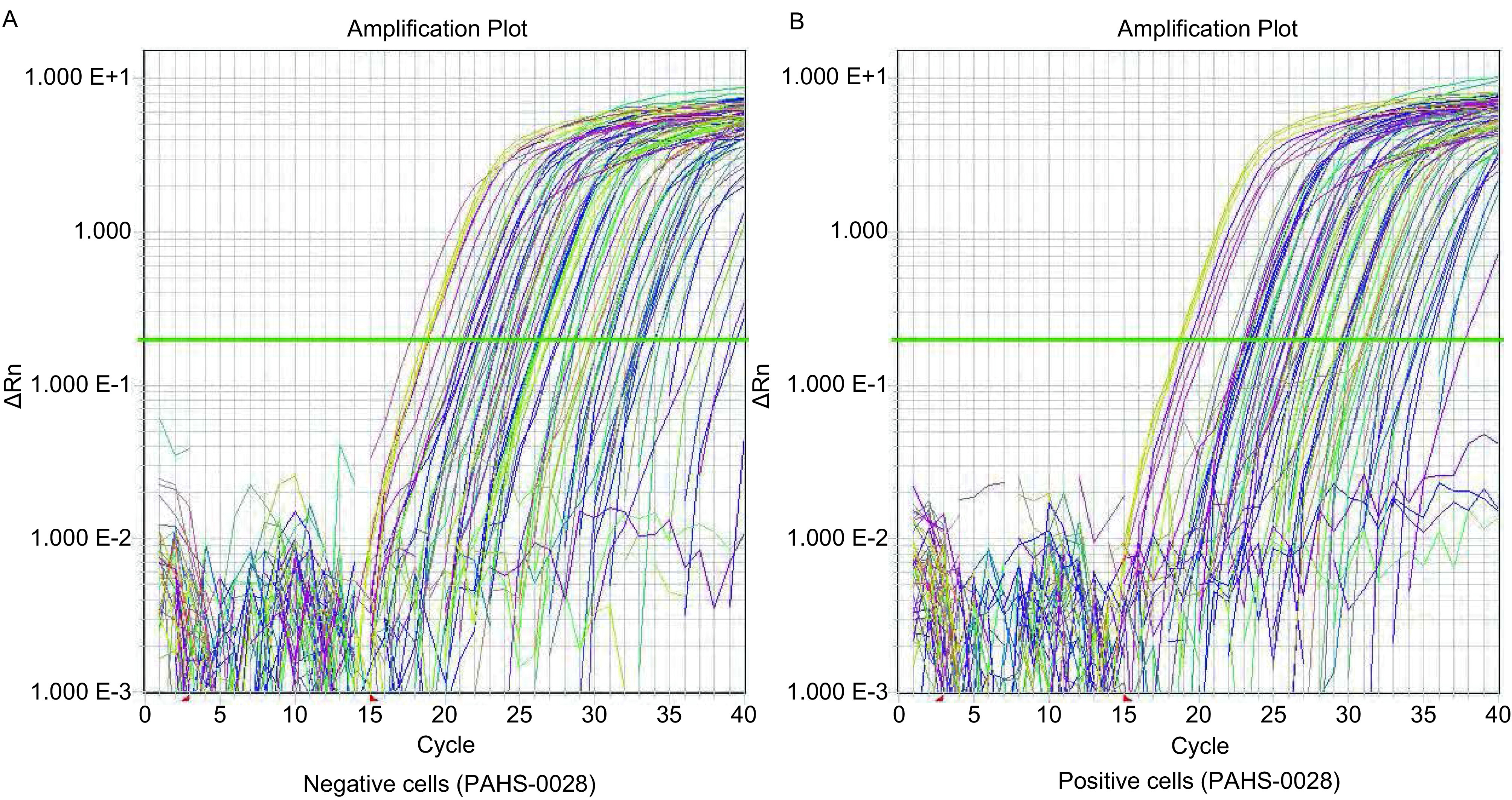
转移基因荧光定量PCR扩增曲线。A：CD133阴性细胞；B：CD133阳性细胞。 Quantitative PCR amplification of metastasis-related genes. A: CD133-negative cells; B:CD133-positive cells.

**3:** CD133+与CD133-细胞的肿瘤转移相关差异基因 Differentially expressed metastasis-related genes between CD133+ and CD133- cells

Gene symbol	Fold change
*CD82*	-12.0
*MMP2*	-4.2
*IL*-*1B*	-3.4
*MYC*	-3.1
*VEGFA*	-3.1
*HTATIP2*	-2.8
*TP53*	-2.6
*ITGB3*	-2.5
*FGFR4*	-2.5
*APC*	-2.5
*MET*	-2.4
*CTBP1*	-2.3
*CDH1*	-2.3
*NF2*	-2.3
*EWSR1*	-2.2
*HGF*	-2.2
*CDH11*	-2.1
*CXCR4*	-2.1
*NME1*	-2.1
If the fold change is positive, it means up-regulation. If the fold change is negative, it means down-regulation. CD82: CD82 molecule; MMP2: matrix metallopeptidase 2; IL1*β*: interleukin 1*β*; MYC: V-myc myelocytomatosis viral oncogene homolog; VEGFA: vascular endothelial growth factor A; HTATIP2: HIV-1 Tat interactive protein 2; TP53: tumor protein p53; ITGB3: integrin, beta 3; FGFR4: fibroblast growth factor receptor 4; APC: adenomatous polyposis coli; MET: met proto-oncogene; CTBP1: C-terminal binding protein 1; CDH1: E-cadherin; NF2: neurofibromin 2; EWSR1: ewing sarcoma breakpoint region 1; HGF: hepatocyte growth factor; CDH11: OB-cadherin; CXCR4: Chemokine, C-X-C motif, receptor 4; NME1: non-metastatic cells 1.	

## 3 讨论

CD133，又称prominin-1/AC133抗原，是prominin家族成员之一。最初，其作为成体干细胞的标志物被大多数学者认识和接受。随后，在包括脑肿瘤、甲状腺癌、乳腺癌、肝细胞癌、胰腺癌、结肠癌、前列腺癌、卵巢癌等肿瘤中发现CD133阳性的肿瘤细胞具有干细胞特性，因而CD133被认为是通用的CSCs表面标记物。为了解CD133在肺癌组织中的表达情况，我们收集了81例人肺癌组织石蜡标本进行IHC染色。在81例肺癌组织中，43例肿瘤细胞CD133呈阳性表达，但阳性细胞数量稀少，余38例肿瘤组织未见CD133阳性细胞。这一结果与Eramo^[6]^的报道有所不同。Eramon采用19例新鲜肺癌组织进行流式细胞术（fluorescence-activated cell sorting, FACS）检测，发现CD133阳性细胞的比例虽然很低，但在所有的标本中都存在CD133阳性细胞。我们认为造成这一差异的原因可能由于：①本研究所用的材料是石蜡包埋组织而非新鲜组织；②Eramon进行FACS所用的新鲜肺癌组织体积较大，而本研究用于IHC的石蜡组织只有3 µm-4 µm厚，仅仅是肿瘤组织中极小的一部分，而众所周知CSCs数量稀少，在肿瘤组织中也只占极低的比例，因此，不能完全认为其余的38例组织中没有CD133阳性细胞。在48例表达CD133的肺癌组织中，CD133阳性的肿瘤细胞呈小灶状/巢状分布。这与以前的发现^[6, 7]^一致，有学者认为这一现象与干细胞“niche”有关。干细胞“niche”是干细胞居留的微环境，正常情况下干细胞聚居于组织器官特定的“niche”中，CSCs因保留了干细胞的特性而呈现“niche”分布现象。我们推测CD133阳性细胞在肺癌组织中呈灶状或巢状分布并非偶然，可能与其具有CSCs特性有关。统计分析CD133表达与肺癌组织学类型、肿瘤的分级及临床分期的关系，证明均无相关性，这与报道的CSCs标记物在肿瘤组织中的表达与肿瘤病理学指标无关相一致^[8]^。

在本研究中，我们采用MACS技术对人肺腺癌A549细胞株中的CD133阳性细胞和CD133阴性细胞进行分选。在分选前，为明确A549细胞株中是否存在CD133阳性细胞，我们对A549细胞株进行IHC染色，结果显示A549细胞株存在少量的CD133阳性细胞。随后，我们对细胞进行分选，结果显示5×10^6^个分选前细胞经过分选获得CD133阳性细胞数约为1×10^4^个，CD133阳性细胞在人肺腺癌A549细胞株中所占比例极低，仅约为0.2%。我们采用荧光素（PE）标记的CD133-2抗体对分选所得细胞进行纯度评估。荧光显微镜下观察经分选所得的CD133阳性细胞可见细胞发明亮的红色荧光；CD133阴性细胞中则仅见极个别发红色荧光的细胞，考虑可能为分选过程中洗脱的阳性细胞或抗体非特异性结合显色。由此可见，采用MACS技术能获得高纯度的CD133阳性细胞和CD133阴性细胞。

对分选所得的CD133阳性/阴性细胞进行生物学特性鉴定主要依据CSCs所具有的特性^[9-11]^，即：①高致瘤性：少于104个甚至是100个肿瘤干细胞就能在免疫缺陷动物体内致瘤；②自我更新特性：肿瘤干细胞能在无血清培养基中悬浮生长并形成“sphere”；③分化潜能：在无血清培养基中悬浮生长所得的“sphere”可被血清诱导分化，呈现贴壁生长^[6]^；④耐药性（drug resistance）；⑤高增殖潜能。在本研究中，我们主要从肿瘤细胞的自我更新特性、分化潜能、增殖潜能三方面对分选所得的CD133阳性/阴性人肺腺癌A549细胞进行比较和鉴定。我们的研究显示：①CD133阳性细胞组在培养的第9天可见“sphere”形成，随培养时间迁移，数量逐渐增多，而CD133阴性细胞组培养至第8周未见“sphere”形成；②CD133阳性细胞组形成“sphere”置于含血清培养基中培养，培养两周全部贴壁。显微镜下观察这些贴壁生长细胞的形态与未经分选的人肺腺癌A549细胞无明显差异。诱导分化前细胞表达CD133、不表达CK7，诱导分化后CD133由阳性转变为阴性，而分化标志物CK7则由阴性转变为阳性。③CD133阳性、CD133阴性、未分选细胞的平均克隆形成率分别为57.3%、3.3%、8.7%，表明三组细胞增殖能力CD133阳性细胞 > 未分选细胞 > CD133阴性细胞。未分选细胞的增殖能力居于二者之间可能与未分化细胞中含少量CD133阳性细胞有关。综上所述，CD133阳性细胞与CD133阴性细胞在自我更新特性、分化潜能、增殖潜能方面具有明显的差异。CD133阳性细胞具有自我更新能力，可被血清诱导分化，且具有更高的增殖潜能。因此，我们认为本实验经MACS获得的CD133阳性人肺腺癌A549细胞具有CSCs的特性。

CSCs被认为是肿瘤转移发生的主要原因，理论上说其在转移相关基因的表达上应该与非肿瘤干细胞有明显的差异。因此，本研究采用基因芯片技术对分选后未经培养的CD133阳性细胞和CD133阴性细胞的肿瘤转移相关基因进行检测，筛选差异基因。我们采用了美国SuperArray公司生产的第二代功能分类基因芯片。该芯片按照功能或信号通路对基因进行了筛选和分类，避免了高通量基因芯片因数据过多而难以分析的缺点。同时，它还将检测精度提高到了与实时定量PCR相当的水平，芯片检测后无需再进行PCR验证。因此，使用该芯片能高效、准确地对CD133阳性细胞和CD133阴性细胞的肿瘤转移基因进行筛选。

在检测的84个转移基因中，差异基因涉及：①促进血管新生的基因（*MYC*、*VEGFA*、*FGFR4*、*HGF*、*MET*、*CXCR4*）和抑制血管生成的基因（*HTATIP*、*TP53*）；②促进细胞迁移的基因（*ITGB3*、*CTBP1*、*APC*、*HGF*、*CXCR4*）和抑制细胞运动的基因（*NF2*、*NME*）；③促进细胞外基质降解的调控因子相关基因（*MYC*、*MMP2*、*ITGB3*、*IL1B*）；④调控细胞间黏附作用的基因（*CDH1*、*CDH11*）；⑤机制未明的基因*CD82*、*EWSR1*。与CD133阴性细胞相比，CD133阳性细胞相关基因表达下调，由此可见，CD133阳性细胞在细胞外基质降解、形成新生血管及细胞运动方面可能并不具备比CD133阴性细胞更高的潜能。我们推测，CD133阳性细胞具有高增殖能力，可通过不断增殖产生大量的子代细胞，这些子代细胞中大部分是CD133阴性细胞，它们高表达正向调控细胞外基质降解、血管新生、淋巴管生成相关的基因，通过合成相应的细胞因子降解细胞外基质、促使血管新生和淋巴管新生，为CD133阳性细胞发生转移营造有利的环境。CD133阳性细胞由于低表达细胞间黏附分子，更易于从原发灶脱落形成“肿瘤芽”进而发生转移。Zlobec等^[12]^的研究也发现在转移性结直肠癌浸润前缘发生上皮细胞间充质细胞化（epithelial-to-mesenchymal transition, EMT）的肿瘤细胞以“肿瘤芽”形式侵润性生长，而这些“肿瘤芽”高度表达CSCs标记物。

在检测的84个肿瘤转移基因中，CD133阳性细胞与CD133阴性细胞在CD82基因的表达差异最为明显。CD82，又称KAI1，是1995年由Dong^[13]^发现并分离的肿瘤转移抑制基因。研究^[14, 15]^表明CD82能有效抑制肺癌的转移。CD82通过与Duffy抗原趋化因子受体（duffy antigen receptor for chemokines, DARC）的相互作用使进入血液循环的CD82+肿瘤细胞与表达DARC的血管内皮细胞发生黏附从而阻断肿瘤细胞的转移^[16]^；它还可能通过抑制转移部位肿瘤细胞的生长从而发挥抑制肿瘤转移的作用^[17]^。我们的研究结果显示，与CD133阴性细胞相比，CD133阳性细胞中CD82的表达降低，两者之间的差异达12倍。我们推测，CD133阳性细胞由于低表达CD82，与DARC+血管内皮细胞的相互作用较弱，易于发生转移；到达转移部位后受CD82抑制增殖的影响较小，更容易形成转移瘤，因此在肿瘤转移过程中发挥关键作用。

迄今为止，关于CSCs中转移相关基因的研究及报道很少，CSCs在肿瘤转移过程中的作用机制仍不清楚，有待进一步研究。
